# Assessment of the Effectiveness of Extracorporeal Shock Wave Therapy (ESWT) For Soft Tissue Injuries (ASSERT): An Online Database Protocol

**Published:** 2014-04-08

**Authors:** G Maffulli, S Hemmings, N Maffulli

**Affiliations:** 1. Curis Consulting Ltd, Fergusson House, 124-128 City Road, London. EC1V 2NJ United Kingdom; 2. Centre for Sports and Exercise Medicine, Queen Mary University of London, Barts and the London School of Medicine and Dentistry, Mile End Hospital, London, E1 4DG United Kingdom; 3. Department of Musculoskeletal Medicine and Surgery, University of Salerno School of Medicine and Surgery, Salerno, Italy

**Keywords:** Chronic soft tissue injuries, extracorporeal shockwave therapy, effectiveness, short and long-term effects

## Abstract

**Background:**

Soft tissue injuries and tendinopathies account for large numbers of chronic musculoskeletal disorders. Extracorporeal shockwave therapy (ESWT) is popular, and effective in the management of chronic tendon conditions in the elbow, shoulder, and pain at and around the heel.

**Methods/Design:**

Ethical approval was granted from the South East London Research Ethics Committee to implement a database for the Assessment of Effectiveness of Extracorporeal Shock Wave Therapy for Soft Tissue Injuries (ASSERT) to prospectively collect information on the effectiveness of ESWT across the UK. All participants will give informed consent. All clinicians follow a standardised method of administration of the ESWT. The primary outcome measures are validated outcome measures specific to the condition being treated. A Visual Analogue Score for pain and the EuroQol will be completed alongside the condition specific outcome tool at baseline, 3, 6, 12 and 24 months post treatment.

**Discussion:**

The development of the ASSERT database will enable the evaluation of the effectiveness of ESWT for patients suffering from chronic conditions (plantar fasciopathy, tennis elbow, Achilles tendinopathy, greater trochanter pain syndrome and patellar tendinopathy). The results will aid the clinicians in the decision making process when managing these patients.

## Background

Soft tissue injuries and tendinopathies are common, and account for a large number of chronic musculoskeletal disorders Tendinopathy is a common debilitating condition^1^ which presents a challenge to clinicians and therapists^2^. The most common tendinopathies involve the Achilles tendon, medial and lateral epicondyles, patellar tendon and the rotator cuff, and patients may endure pain and disability for years^3^,^4^.

Shock wave therapy (SWT) has been successfully employed since the late 1980s for the management of various musculoskeletal, disorders including plantar fasciopathy, Achilles tendinopathy, shoulder calcific tendinitis, and lateral epicondylitis^5^. Some trials have evidenced negative results 6,7,8,9, but there are now many well performed randomized, double-blind, clinical trials which support the use of SWT^10^,^11^,^12^,^13^,^14^,^15^,^16^,^17^,^18^,^19^,^20^. Extracorporeal shockwave therapy (ESWT) is effective in the management of a number of chronic tendon conditions in the elbow, shoulder, and pain at and around the heel. When other forms of conservative therapy have not been effective in relieving pain and other symptoms of tendinopathy, ESWT has been used to relieve pain and improve function.

Despite the growing numbers of published studies on ESWT supporting its use, there is still uncertainty around the use of ESWT and its clinical effectiveness remains controversial. Further research needs to be undertaken, utilising a homogenous intervention; identical outcome assessment; comparable participants and comparable follow-up evaluation for all the conditions for which ESWT is used as a treatment modality^21^. The National Institute for Health and Clinical Excellence (NICE) has published guidance on the use of ESWT in calcific tendinopathy of the shoulder^22^, recommending that the results of the procedure are monitored, and clinicians undertaking the procedure make special arrangements for audit. For plantar fasciitis 23, tennis elbow^24^ and Achilles tendinopathy 25, NICE recognise that the procedure is efficacious in selected patients, and therefore has the potential for a high clinical impact. This makes provision of robust data particularly important; NICE requests that clinicians undertaking the procedure make special arrangements for audit. For greater trochanter pain syndrome 26, NICE identified that 70% of patients would recommend this procedure to others, and requires that clinicians undertaking the procedure make special arrangements for audit. For patellar tendinopathy, there is no NICE guidance, but ESWT in patellar tendinopathy ‘seems to be a safe and promising treatment’ 27.

The primary aim of the Assessment of Effectiveness of Extracorporeal Shock Wave Therapy (ESWT) for Soft Tissue Injuries (ASSERT) database is to determine the effectiveness of ESWT in patients suffering from refractory plantar fasciopathy, tennis elbow, Achilles tendonopathy, greater trochanter pain syndrome, patellar tendinopathy in both the short and long term.

## Methods/Design

An online database (ASSERT) has been developed and implemented to collect information on the effectiveness of ESWT across the UK. A standard ESWT machine (Swiss Dolorclast) and a standardised treatment protocol, together with standardised baseline measurements and outcome measures and time points in centres across the UK have been adopted to aid validity.

## Recruitment

Participants will be recruited from (both the NHS and private sectors) centres in the UK where have the Swiss Dolorclast Shockwave Machine (EMS Ch. de la Vuarpillière 31, 1260 Nyon Switzerland) is used. Clinicians will recruit participants presenting with one of the refractory conditions as indicated above and for whom ESWT has been indicated as a treatment choice.

## Participants

Participants will be included if they are over the age of 16 and have:

- A diagnosis of plantar fasciopathy; tennis elbow; Achilles tendinopathy; greater trochanter pain syndrome; or patellar tendinopathy which has been confirmed by a recruiting clinician and validated by MRI scan or ultrasound scan with power Doppler
- Underwent a course of conservative therapy which has not been effective in relieving pain and other symptoms
- Been recommended to receive ESWT at one of the identified centres
- No inflammatory arthropathies
- Demonstrated the ability to give informed consent.

## Use of ESWT Machine

Standardisation of the machine and the process of administration of ESWT has been agreed to ensure consistency, reproducibility and generalisability of the results. All clinicians using the Swiss Dolorclast machine will receive training and certification to ensure adherence to the protocol. All clinicians follow the Gerdesmeyer method. This includes delivering an initial 500 sensitising impulses at a low air pressure (1.5 bar of air pressure) which reduces the pain the patient experiences during the treatment. Based on patient feedback, the clinician increases the air pressure to 2.5 bar or above. Overall, irrespective of the area being treated, the total dose of impulses remains constant at 2500 per session, with one session a week for 3 consecutive weeks, with a maximum gap between treatments of 2 weeks.

## Database

The ASSERT database is a web based system (www.assert.org.uk) from which the clinician receives a study number for each participant. Only unidentifiable information with the patients study number is entered into the database. Sensitive data is held on secure servers.

Following informed consent, the clinician records the following information:

- Diagnosis as diagnosed by an MRI scan or ultrasound scan with power or colour Doppler (relevant images to be imported onto a JPeg for review)
- Area to be treated/condition presenting with
- Date of presentation of symptoms
- Date of treatment of ESWT
- Code for clinicians centre
- Centre where treatment is administered
- Record any other forms of treatment have been administered prior to consultation and what treatment
- Side the treatment is being administered (Right/Left/Bilateral)
- Dates ESWT administered
- Baseline score recorded: EuroQol (EQ-5D)^28^ and VAS for pain and validated outcome measures according to condition (listed in the outcome measures section)
- Follow-up scores at 3, 6, 12 and 24 months post treatment using validated scoring systems according to the condition
- Visual Analogue Scale (VAS) for pain
- Satisfaction (rated poor, satisfactory, good or excellent)
- Time to effective treatment
- Recurrence of condition
- Complications
- Adverse events

## Arrangements for monitoring the database’s systems and procedures

A data monitoring committee has been formed, consisting of members of the study team, including a lay member and an independent chair, who will meet on a regular basis to review the progress of ASSERT and database management.

## Data collection pilot

Prior to commencement of the data collection, a pilot test with three nominated clinicians and two lay members tested the feasibility of using the database to collate information on patients having ESWT. All recruiting clinicians have to complete the mandatory training to ensure they are both competent to administer the ESWT with the Swiss Dolorclast Machine and they are fully conversant with GCP guidelines and competent with data entry.

## Baseline Assessments

Baseline assessments will be undertaken by the treating clinician following informed consent

## Follow-up Assessments

Outcomes will be assessed at baseline, 3,6,12 and 24 months post treatment. The clinician will undertake the 3 months assessment and the co-ordinators of ASSERT will undertake all other assessment via telephone or post.

## Outcome Assessment

For all conditions the VAS and the EuroQol^28^ will be completed alongside the condition specific outcome tool. VAS is a horizontal line, 100 mm in length asking the patient “How severe is your pain today?” and to indicate on the line how bad their pain is on that day, with one end being “no pain” and the other being “very severe pain”. The EQ-5D^28^ is a standardised measure of health status developed by the EuroQol Group to provide a simple, generic measure of health for clinical and economic appraisal. It is a simple questionnaire designed for completion by the person being treated. The primary outcome measures are specific to the condition being treated and are listed below:

## Outcome Measures

### Calcific tendinopathy of the shoulder

The Upper Extremity Function Scale^29^ is a self-administered questionnaire which can be used to measure the impact of upper extremity disorders on a person's ability to perform physical tasks. It can be used to monitor the patient over time both to detect worsening of the condition and to assess response to a therapeutic intervention.

## Plantar Fasciopathy

The Foot Function Score^30^ is a specific foot function score which provides information as to how foot pain has affected a persons ability to manage in every day life.

## Tennis Elbow

The Patient Rated Tennis Elbow Evaluation (PRTEE)^31^ is a 15-item questionnaire designed to measure forearm pain and disability in patients with lateral epicondylitis (also known as “tennis elbow”). The PRTEE allows patients to rate their levels of tennis elbow pain and disability.

## Achilles Tendinopathy

The VISA-A^32^ is an index of severity of Achilles tendinopathy. The continuous numerical result of the VISA-A questionnaire has the potential to provide utility in both the clinical setting and research. The VISA-A score can identify patients who need more aggressive management, and can be used to monitor their progress.

## Greater Trochanter Pain Syndrome

The Lower Extremity Functional Score^33^. The Lower Extremity Functional Scale (LEFS) can be used to evaluate the functional impairment of a patient with a disorder of one or both lower extremities. It can be used to monitor the patient over time and to evaluate the effectiveness of an intervention.

## Patellar tendinopathy

The VISA-P Outcome Score^34^ is a questionnaire developed for patients with patellar tendinopathy. Patients assess severity of symptoms, function and ability to participate in sport.

The two-year follow-up period will ensure a long enough follow-up to time to assess the treatment effects, since the metabolic turnover rate of tendon tissue is slow^35^

## Adverse events

These will be recorded throughout the whole data collection period.

## Statistical analysis

Descriptive and comparison statistics will be performed on all variables using the SPSS-19 statistics package.

## Ethics

ASSERT will take place in centres across the UK both in the NHS and the private sectors following the principles of the Helsinki declaration and following Good Clinical practice Guidelines (GCP). Ethical approval for ASSERT was granted from the South East London NRES committee (REC Ref: 11/LO/0253). Participants will receive information about ASSERT before providing their written consent. All data collected will be confidential.

## Discussion

At present, ESWT is only used in few centres, and the effectiveness of the treatment being monitored in a non consistent manner. There is no UK database which monitors the efficacy of the treatment in the formal manner as recommended by NICE. The development of this database will allow documentation of the recruitment and outcome of ESWT in a scientific fashion. Collecting information in a pragmatic and yet systematic way via the ASSERT database will ensure the evaluation of the effectiveness of this already established treatment both in the short and long term and will facilitate its use in the wider community.

## List of Abbreviations

ASSERT: ssessment of the Effectiveness of Extracorporeal Shock Wave Therapy for Soft Tissue Injuries
ESWT: Extracorporeal Shock Wave Therapy
NICE: National Institute of Clinical Excellence
VAS: Visual Analogue Score
EQ-5D: EuroQol Quality of Life Outcome Measure
PRTEE: Patient Related Tennis Elbow Evaluation
VISA-A: Victorian Institute of Sport Assessment-Achilles Questionnaire
VISA-P: Victorian Institute of Sport Assessment-patellar Questionnaire
